# Modulation of Gene Expression in Actinobacteria by Translational Modification of Transcriptional Factors and Secondary Metabolite Biosynthetic Enzymes

**DOI:** 10.3389/fmicb.2021.630694

**Published:** 2021-03-16

**Authors:** Juan F. Martín, Paloma Liras, Sergio Sánchez

**Affiliations:** ^1^Área de Microbiología, Departamento de Biología Molecular, Universidad de León, León, Spain; ^2^Instituto de Investigaciones Biomédicas, Universidad Nacional Autónoma de México, México, Mexico

**Keywords:** actinobacteria, post-translational modifications, protein phosphorylation, protein acetylation/acylation, acyl-AMP forming enzymes, synthetases *versus* synthases, transcriptional factors, phosphopante- theneylation

## Abstract

Different types of post-translational modifications are present in bacteria that play essential roles in bacterial metabolism modulation. Nevertheless, limited information is available on these types of modifications in actinobacteria, particularly on their effects on secondary metabolite biosynthesis. Recently, phosphorylation, acetylation, or phosphopantetheneylation of transcriptional factors and key enzymes involved in secondary metabolite biosynthesis have been reported. There are two types of phosphorylations involved in the control of transcriptional factors: (1) phosphorylation of sensor kinases and transfer of the phosphate group to the receiver domain of response regulators, which alters the expression of regulator target genes. (2) Phosphorylation systems involving promiscuous serine/threonine/tyrosine kinases that modify proteins at several amino acid residues, e.g., the phosphorylation of the global nitrogen regulator GlnR. Another post-translational modification is the acetylation at the epsilon amino group of lysine residues. The protein acetylation/deacetylation controls the activity of many short and long-chain acyl-CoA synthetases, transcriptional factors, key proteins of bacterial metabolism, and enzymes for the biosynthesis of non-ribosomal peptides, desferrioxamine, streptomycin, or phosphinic acid-derived antibiotics. Acetyltransferases catalyze acetylation reactions showing different specificity for the acyl-CoA donor. Although it functions as acetyltransferase, there are examples of malonylation, crotonylation, succinylation, or in a few cases acylation activities using bulky acyl-CoA derivatives. Substrates activation by nucleoside triphosphates is one of the central reactions inhibited by lysine acetyltransferases. Phosphorylation/dephosphorylation or acylation/deacylation reactions on global regulators like PhoP, GlnR, AfsR, and the carbon catabolite regulator glucokinase strongly affects the expression of genes controlled by these regulators. Finally, a different type of post-translational protein modification is the phosphopantetheinylation, catalized by phosphopantetheinyl transferases (PPTases). This reaction is essential to modify those enzymes requiring phosphopantetheine groups like non-ribosomal peptide synthetases, polyketide synthases, and fatty acid synthases. Up to five PPTases are present in *S. tsukubaensis* and *S. avermitilis*. Different PPTases modify substrate proteins in the PCP or ACP domains of tacrolimus biosynthetic enzymes. Directed mutations of genes encoding enzymes involved in the post-translational modification is a promising tool to enhance the production of bioactive metabolites.

## Introduction: Post-Translational Modification of Proteins Plays an Important Role in Cell Metabolism

During the last decades, numerous records indicated that protein modifications play a key role in enzyme activities, protein–protein interactions or DNA–protein recognition, and signaling. Post-translational modifications (PTM) include phosphorylation of proteins at histidine, serine, threonine or tyrosine residues, acylations, methylations, phosphopantetheinylations, glycosylations, ubiquinations, and proline isomerization, among others. Post-translational modifications were initially studied in eukaryotic organisms, e.g., the eukaryotic histones were the first well studied acylated proteins (Waterborg, 2001; Sabari et al., 2017); also studies in eukaryotes showed that acylation affected important transcriptional factors (Boyes et al., 1998; Marzio et al., 2000). These studies were later expanded to a large number of different bacteria. However, only in the last decade, there have been important studies on post-translational modification of proteins in *Streptomyces* and related actinobacteria. These modifications, particularly phosphorylation, acylation at lysine residues and phosphopantetheinylation, are very important to understand the control of metabolism in this group of bacteria which are prolific producers of a variety of secondary metabolites (also named specialized metabolites). There is increasing evidence than acylation of proteins at lysine residues plays a significant role in the regulation of the biosynthesis of secondary metabolites in actinobacteria. Acetylation, propionylation, and malonylation are the more frequent lysine modification of enzymes, although crotonylation and succinylation have also been reported. In this article we focus on the post-translational modification of biosynthetic enzymes and of transcriptional factors that control expression of genes. We do not intend to cover all post-translational modifications of proteins of actinobacteria, but only those known to affect the expression of genes or the activity of key enzymes involved in the biosynthesis of secondary metabolites.

## Protein Phosphorylation in Bacteria: The Phosphoproteome

Protein phosphorylation is a mechanism to control the activity of many proteins in bacteria. The extent of protein phosphorylation in prokaryotes is lower than in eukaryotes. In *Streptomyces* and related actinobacteria, the modification processes have been less studied. Particularly relevant is the phosphorylation of transcriptional factors and other regulatory proteins.

Two major types of post-translational phosphorylations modify proteins of the *Streptomyces* genus and other Gram-positive bacteria. The first class includes the phosphorylation/dephosphorylation reactions by the Two Component Systems (TCS) PhoR sensor kinase (Hutchings et al., 2004; Martín et al., 2012). A second class includes protein phosphorylation exerted by promiscuous serine/threonine/tyrosine kinases (STK) that phosphorylate amino acid residues containing a free hydroxyl group.

### Phosphorylation Cascades in Two Component Systems Modify a Variety of Transcriptional Factors

The TCSs include a sensor histidine kinase, and a response regulator and the phosphorylation by these systems is extremely site-specific (Mascher et al., 2006; Gross and Beier, 2012).

The sensor kinases are usually membrane-embedded proteins able to self-phosphorylate at a conserved histidine residue (His^165^ in *Streptomyces coelicolor* and *Streptomyces lividans* PhoR) in response to either an extracellular or an intracellular signal. The sensor kinases studied in streptomycetes have an intracellular amino acid span (50–500) bordered at the N-terminal region by two to six transmembrane domains ending in an amino acid loop, normally intracellular. These proteins contain a transmembrane domain at the C-terminal end, with an extracellular chain of about 60 amino acids (McKenzie and Nodwell, 2009). The phosphorylated form of the sensor kinase has a short life because, in addition to its autokinase activity, it also has phosphatase activity. The phosphorylated histidine and the amino acids involved in the phosphatase activity are located in the intracellular span (e.g., in the AbsAB1 sensor kinase at His^202^ and L^253^, respectively). The stimulus signal modifies the C-terminal structure, resulting in histidine-autophosphorylation, which initiates the cascade of regulation. In the TCSs sensor kinases, the phosphate group at the phosphorylated histidine is transferred to an aspartate residue of the cognate response regulator, which interacts in its phosphorylated state with the promoter of genes regulated by the TCS. In the absence of an external signal, the phosphorylated histidine kinase loses its ability to transfer the phosphate group (Kalantari et al., 2015).

The response regulators (RR) have a receiver domain containing a conserved aspartate. The typical receiver pocket is well conserved in response regulators, e.g., in *Escherichia coli* OmpR includes the amino acids D^11^, D^12^, D^55^, T^87^, K^105^, and Y^106^, being D^55^ the phosphorylated amino acid (Brissette et al., 1991; Lin et al., 2014). Phosphorylation of this amino acid residue results in activation of an effector domain in the carboxy terminal region containing a DNA-binding domain (DBD), which elicits the physiological response.

The phosphorylated response regulator is dephosphorylated by the specific phosphatase activity of the sensor kinase, thus allowing the modulation of the phosphorylation state of the response regulator and, hence, its ability to regulate the transcription of the target operons. At the cytoplasm neutral pH, the half-life of the phosphorylated aspartate residue in the response regulator is very short.

### PhoP as a Model for Phosphorylation of Response Regulators

The PhoR-PhoP TCS is a model system in *Streptomyces* that controls the global phosphate metabolism and many secondary metabolites biosynthetic clusters. The PhoR sensor kinase responds to a signal that detects phosphate concentrations (Martín and Liras, 2020). The PhoP response regulator has 223 amino acids and belongs to the OmpR family. The amino acid sequence of all the *Streptomyces* PhoP proteins is highly conserved (90–99% identity); they always contain the amino acids D^6^, D^49^ for phosphorylation, and K^98^. In the carboxy-terminal region, PhoP has a DNA-binding motif (amino acids 190–201) for the control of transcription of the PhoP regulated genes.

The phosphorylation cascade in the PhoP-PhoR system has been extensively studied and reviewed (Sola-Landa et al., 2003, 2005; Rodríguez-García et al., 2007; Martín-Martín et al., 2018; Martín and Liras, 2020) and therefore, is not described in detail here.

### Protein Phosphorylation by Promiscuous Serine/Threonine/Tyrosine Kinases

In contrast to the TCS specific sensor phosphorylation of the TCS response regulator, promiscuous Hanks-type serine/threonine kinases (STK) might phosphorylate different proteins, including TCS sensor kinases, TCS response regulators, and other transcriptional regulators (Wright and Ulijasz, 2014; Kalantari et al., 2015; Mijakovic and Deutscher, 2015).

The phosphorylated serine/threonine/tyrosine has a longer half-life than the aspartate-phosphorylation of the TCSs, allowing the regulation in *Mycobacterium tuberculosis* of multiple cellular functions such as stress response, cell-wall modifications, or antibiotics resistance (Wright and Ulijasz, 2014).

In *Streptomyces*, the phosphoproteome was initially studied by Parker et al. (2010) and Manteca et al. (2011) and later by Ladwig et al. (2015) and Rioseras et al. (2018). In *S. coelicolor*, the relative levels of phosphorylation of Ser, Thr, and Tyr residues are 34, 52, and 14%, respectively. Phosphorylation of these sites plays a role in regulating essential aspects of metabolism in streptomycetes. The number of phosphorylated regulators identified in *Streptomyces* is larger than in other bacterial phosphoproteomes, which may be explained by the nutritional variability of *Streptomyces* and its complex developmental life cycle (Parker et al., 2010). The phosphoproteome of *S. coelicolor* revealed 85 protein phosphorylation sites (Rioseras et al., 2018). In the same study, 48 phosphoproteins were quantified and showed significant variation throughout *S. coelicolor* development stages. Phosphorylation of the cell division proteins FtsZ and DivIVA, and of other proteins at Ser/Thr/Tyr sites appears to be important in modulating the sporulation initiation and the formation of secondary metabolites (Manteca et al., 2011; Rioseras et al., 2018).

### Phosphorylation of the Global Nitrogen Regulator GlnR in *Streptomyces* by Promiscuous Kinases

Some response regulators are orphan (i.e., they lack the cognate sensor kinase) and may lack some of the amino acids lining the receiver domain pocket (Lin et al., 2014). These types of orphan regulators are phosphorylated by promiscuous serine or threonine kinases as occurs with the atypical GlnR orphan regulator. GlnR controls nitrogen metabolism in *S. coelicolor* and other *Streptomyces* species. The unphosphorylated GlnR protein binds to the promoter regions of *glnA, glnII, amtB*, *nirB, glnR*, and other genes involved in nitrogen metabolism (Tiffert et al., 2008; Rodríguez-García et al., 2009; Amin et al., 2012; Sola-Landa et al., 2013). A full expression of the GlnR targeted genes occurs when the GlnR regulator is unphosphorylated (Amin et al., 2016).

GlnR has an aspartate residue (D^50^) in the putative receiver domain but lacks other conserved amino acids in the receiver pocket (Lin et al., 2014). In polyacrylamide gel electrophoresis the GlnR protein shows two different forms of 35 and 38 kDa. These forms can be detected with anti-GlnR antibodies, indicating a post-translational modification of the protein. In a nitrogen-rich medium, phosphorylation of GlnR occurs in six serine and threonine sites, located in the DNA binding region of GlnR; noteworthy, the degree of phosphorylation modulates the binding to the target promoters. In nitrogen-limited defined medium, only two of those residues are phosphorylated but no phosphorylation occurs in nitrogen-poor defined medium (Amin et al., 2016). These observations indicate that post-translational modification of GlnR responds to the nitrogen availability either in complex or in defined medium. In summary, phosphorylation of GlnR prevents binding of this global regulator to its targets.

### Modification of AfsR by Phosphorylation

The AfsR is a large protein (933 amino acids) related to the SARP family of *Streptomyces* regulators containing an OmpR-type DNA binding domain at the N-terminal end. In *S. coelicolor*, it is encoded by *afsR*, a gene with orthologs in many *Streptomyces* species.

AfsR indirectly regulates the production of the actinorhodin (Act), undecylprodiginine (Red), and calcium-dependent antibiotic (Cda) biosynthesis in *S. coelicolor* by controlling the expression of the adjacent gene a*fsS* (Horinouchi, 2003). The small protein AfsS (63 amino acids) is an ancillary activator that binds the region upstream of the genes encoding the specific activators of the *act, red*, and *cda* clusters; this allows their expression and the subsequent production of the three antibiotics (Vögtli et al., 1994; Floriano and Bibb, 1996; Lee et al., 2002).

Expression of *afsS* requires a functional AfsR protein. The AfsR protein is phosphorylated at serine/threonine residues by the AfsK kinase and the phosphorylation strongly increases its binding affinity for the *afsS* promoter and the *afsS* expression (Hong et al., 1991; Lee et al., 2002). The *afsS* promoter region has an abnormal distance between the −10 and −35 motifs, and has been proposed that the phosphorylated AfsR bends the DNA backbone at the *afsS* promoter region, thus allowing proper interaction with the RNA polymerase (Horinouchi, 2003). Interestingly, the AfsR binding sequence in the *afsS* promoter is the same sequence recognized by the PhoP global regulator, and there is a binding competition between these two regulators (Santos-Beneit et al., 2009, 2011). The AfsK kinase-mediated transfer of phosphate to AfsR is inhibited by the binding of KbpA, a negative regulator of the AfsK-AfsR phosphorylation cascade, which indirectly decreases secondary metabolism in *S. coelicolor* and also sporulation in *S. griseus* (Umeyama and Horinouchi, 2001; Sawai et al., 2004).

The AfsK–AfsR couple of proteins also act as a sensor system of S-adenosyl methionine (SAM), the methyl donor in methylation reactions (Jin et al., 2011; Martín and Liras, 2020). The findings of the AfsK modification effects indicate that phosphorylation cascades may serve to integrate in the TCSs of *Streptomyces* other regulatory signals.

## Protein Modification by Acetylation of Lysine Residues: The Acetylosome

The classical protein acetylation by the GCN5-type N-acetyl transferases occurs at the amino epsilon group of the lysine residues by a family of enzymes known as lysine acetyltransferases (named KATs or PATs). The epsilon amino group (at carbon-6) of lysine protrudes from the polypeptide skeleton of the protein.

The acetylation of lysine neutralizes the positive charge of this residue resulting in a reorganization of the protein structure (Sabari et al., 2017). It is important to note that the regulation of enzymes by protein acetylation at the lysine residues depends on the degree of acetylation which is the result of two opposite activities, namely an N-acetyltransferase (KAT) and a lysine deacetylase (KAdea) that remove the acetate molecule from the protein. One group of deacetylases requires NAD as a cofactor (Sirtuin-dependent deacylases, named SIRT). The second class of deacetylases (Zn^2+^ dependent amidases) cleaves the amide bond, releasing the acetate group.

Significant advances in the knowledge of protein post-translational acetylation for the control of metabolism, both in prokaryotic and eukaryotic organisms have been achieved during the last decade (Bernal et al., 2014). Early studies on the proteome of Enterobacteria provided information on these bacteria acetylome (Zhang et al., 2009; Wang et al., 2010). Studies on the Gram-positive bacteria *Bacillus subtilis* evidenced a protein lysine acetyltransferase, named AcuA, which modifies the acetyl-CoA synthase (Gardner et al., 2006). These modifications affect key enzymes and regulatory factors, and transcriptional/translational mechanisms in cell metabolism (Choudhary et al., 2014).

### Characteristics of the Protein Lysine Acetyl Transferase in Actinobacteria

**FIGURE 1:**
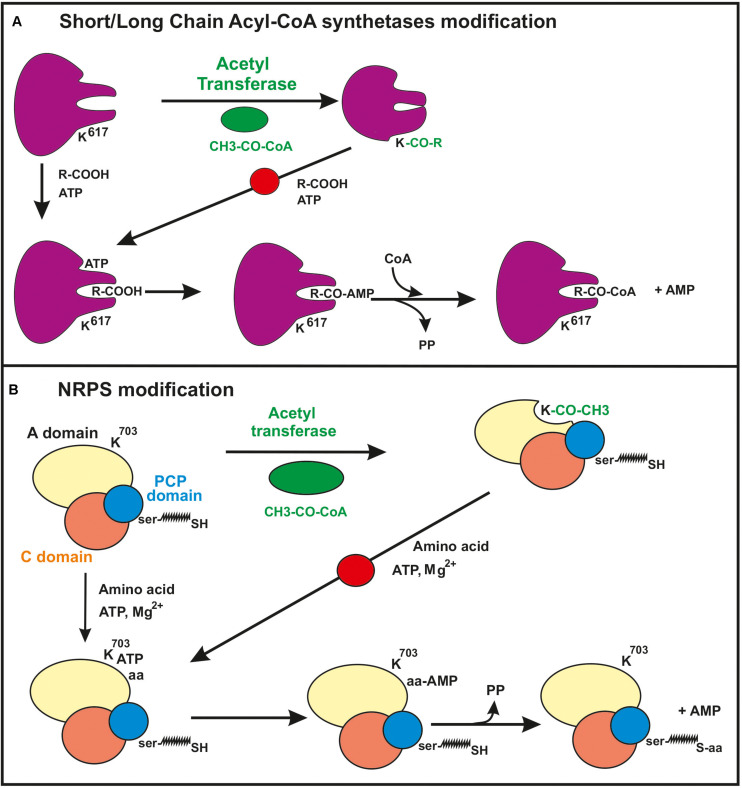
Mechanism of inhibition by acetylation of the first step of the activation reaction of short/long chain fatty acid in acyl-CoA synthetases [panel **(A)** and amino acids in non-ribosomal peptide synthetases (NRPSs) (panel **B**)]. **(A)** In the short/long chain acyl-CoA synthetases a lysine residue (K617) in the acetoacetyl-CoA synthetase protein is acetylated resulting in inhibition of the formation of the acyl-AMP intermediate. **(B)** Inhibition by lysine acetylation of the formation of the aminoacyl-AMP intermediate of NRPSs. The lysine residue in the studied NRPS of *S. roseosporus* corresponds to K^703^. This acetylation prevents the formation of the aminoacyl-AMP (see text for details). The domains A (for amino acid activation), PCP (peptidyl carrier protein) with the phosphopantetheinyl arm and C (condensation domain) of the NRPS are shown. Note the similarity of the molecular mechanisms involved in the modification of acyl-CoA synthetase and NRPSs. The red circle on the arrow indicated that this reaction is blocked by the acylation of the enzyme.

Protein acetylation at the lysine residues occurs also in mycobacteria (Nambi et al., 2010; Xu et al., 2011; Liu et al., 2014), *Streptomyces roseosporus* (Liao et al., 2015) or *Saccharopolyspora erythraea* (Huang et al., 2015). Mikulik et al. (2012) observed that the acetyl-CoA synthetase of *S. coelicolor* is acetylated *in vivo* by a protein lysine acetyltransferase and Tucker and Escalante-Semerena (2013) characterized a gene of *S. lividans* that encodes a large protein acetyl transferase. This protein contains two domains, a 700-amino acid C-terminal region that encodes an NDP-forming CoA synthetase-like protein, and a 200 amino acid GNAT domain in the N-terminal region, an organization reverse with respect to that of homologous enzymes of Enterobacteria. This reverse organization is conserved in other actinobacteria and some archaea. The protein, named PatA, acetylates both the acetyl-CoA synthetase and the acetoacetyl-CoA synthetase, although the acetylation of the second enzyme was 34-fold higher than that of the first one. The *S. lividans* protein acetyl transferase Sl-PatA was expressed and purified from *E. coli* and it was shown to acetylate the acetoacetyl-CoA synthetase at the residue K^617^. Moreover, a SIRT-type deacetylase reverts the acetylation of the acetoacetyl-CoA synthetase. Therefore, acetylation/deacetylation reactions likely modulate the activity in *Streptomyces* and related actinobacteria (Tucker and Escalante-Semerena, 2013). The accumulated evidence from studies with both Gram-positive and Gram-negative bacteria indicates that the acetylation of the substrate enzyme is exerted by inhibiting the first half reaction for activation of short chain fatty acid, i.e., the formation of acyl-AMP (acyl adenylates) by the substrate enzyme (Figure 1). This mechanism is important to understand how acetylation of other enzymes involved in the formation and activation of precursors of polyketide and non-ribosomal peptide synthetases may be affected [see sections “Regulation of the formation of polyketide precursors by protein lysine acetylation” and “Non-ribosomal peptide synthetases (NRPSs)”].

### Control of the Acetyltransferase Activity by Allosteric Effectors

In *M. tuberculosis* and *Mycobacterium smegmatis*, the regulation of the acetyltransferase (PatA) activity is exerted by cAMP that directly binds to a cyclic nucleotide binding domain fused to the N-terminal end of GCN5-type KATs (Nambi et al., 2010). In *Micromonospora aurantiaca*, an amino acid-binding domain fused to the GNAT domain of the acetyltransferase participates in its amino acid allosteric regulation. Bioinformatic analysis revealed many amino acid-sensing acetyltransferases (named AAPatA) (Lu et al., 2017). In all of them, the amino acid-binding domain is close to the GNAT domain, thus conferring sensitivity to allosteric regulation by amino acids. The amino acids asparagine and cysteine are the major allosteric effectors in actinobacteria. In conclusion, it seems that the enzymatic activity of protein acetyltransferases is regulated by the available amino acids and perhaps by other nitrogen sources.

### Regulation by GlnR of the Expression of *AcuA*, Encoding the Lysine Acetyl Transferase, in *Saccharopolyspora erythraea*

Very little is known about the control of expression of acetyltransferase genes in actinobacteria and other Gram-positive bacteria. Expression of the *acuA* gene in *Sac. erythraea* is influenced by nutritional and environmental conditions, particularly by the nitrogen source. Indeed, a GlnR binding site is present in the promoter region of the *acuA* gene in this actinobacterium (You et al., 2014, 2016). This finding was confirmed by electrophoretic mobility assays using purified GlnR. Expression of *acuA* was activated in mutants deleted in the *glnR* gene.

### Specificity of the Lysine Acetyl Transferases in *Streptomyces* and Related Actinobacteria

The lysine acetyl transferase of *Sac. erythraea* usually utilizes acetyl-CoA as a substrate (Lu et al., 2017; Xu Y. et al., 2018). Although acylation of the phosphate global regulator PhoP introduces a propionyl group at the lysine residue, the enzyme should be considered an acyl transferase with wide substrate specificity. Several other lysine acylations, including propionylation, malonylation, succinylation, glutarylation, or crotonylation have been reported in different bacteria (Hirschey and Zhao, 2015) and some of them also in *Streptomyces* and other actinobacteria (Table 1). One important question is whether the same acetyltransferase is responsible for the acylations of proteins using different acyl-CoA molecules particularly the bulky acyl-CoA molecules.

**TABLE 1:** Acylation reactions at lysine residues of key regulatory proteins.

**Acylation mechanism modification**	**Reaction**	**Donor Molecules**	**References**
Two carbons modification	Acetylation	Acetyl-CoA	Huang et al., 2015; Xu J. Y. et al., 2018
Three carbons	Propionylation	Propionyl-CoA	Xu Y. et al., 2018
	Malonylation	Malonyl-CoA	Qian et al., 2016; Xu J. Y. et al., 2016
Four carbons	Butirylation	Butiryl-CoA	Xu J. Y. et al., 2018
	Succinylation	Succinyl-CoA	Weinert et al., 2013; Yang et al., 2015; Mizuno et al., 2016
	Crotonylation	Crotonyl-CoA	Xu et al., 2017
	β-Hydroxybutyrylation	β-Hydroxybutyryl-CoA	Xu J. Y. et al., 2018
	β-Isobutyrylation	β-Isobutyryl-CoA	Xu J. Y. et al., 2018
Five carbon	Glutarylation	Glutaryl-CoA	Hirschey and Zhao, 2015; Xie et al., 2016

Many proteins are crotonylated rather than acetylated in *S. roseosporus.* Using a mutant of *S. roseosporus* deleted in the gene encoding the Kct1 acetyltransferase, Sun et al. (2020a, b) found low protein crotonylation during the mutant growth phase. This finding suggests that this enzyme is partially responsible for the crotonylation reaction; however, there were no differences in the amount of crotonylated proteins during the late differentiation and secondary metabolites production phases between the parental strain and the Kct1 mutant. The deleted Kct1 mutant showed retarded differentiation. Besides, the onset of daptomycin production was greatly delayed, indicating that high levels of lysine modification by this enzyme are essential for the normal production of antibiotic (Sun et al., 2020b). These results suggested that there are other enzymes able to crotonylate *S. roseosporus* proteins different from Kct1. Alternatively, the same acetyl transferase might be able to introduce crotonyl groups at the lysine residue; this is supported by a similar observation in eukaryotic cells (Tan et al., 2011; Sun et al., 2017; Xu et al., 2017).

Other proteins can be modified by protein lysine succinylation. Both enzymes acetylation and succinylation of central metabolism enzymes occurs in *Corynebacterium glutamicum;* an actinobacterium used for industrial glutamic acid and lysine production. The flux of intermediates through the TCA cycle changes during the fermentation favoring the formation of 2-oxoglutarate and glutamic acid. A study of the acetylome and the succinylome of *C. glutamicum* during the glutamic acid fermentation revealed that acetylation of key enzymes decreases, whereas succinylation of these enzymes increases coinciding with the glutamic acid production stage (Mizuno et al., 2016). In this study, up to 604 acetylated proteins and 288 succinylated proteins were identified. Particularly relevant is the post-translational modification of two dehydrogenase complexes. These 2-oxoglutarate dehydrogenase and pyruvate dehydrogenase complexes play a key role in glutamic acid biosynthesis. The protein components of these complexes were both acetylated and succinylated in 42 acylation sites in the different components of both complexes. This finding indicates that modification of these lysine residues plays an important role in regulating the glutamic acid biosynthesis. The correlation between the higher succinylation of the 2-oxoglutarate dehydrogenase complexes and the glutamic acid production suggests a predominant modification by succinylation during the production stage, probably because of the higher availability of succinyl-CoA at that stage of the cell culture.

### Regulation of the Formation of Polyketide Precursors by Protein Lysine Acetylation

The enzymes involved in the formation of polyketide precursors have been characterized in many bacteria, including *Streptomyces.* Standard starters and elongation units for various polyketide synthases (types I, II, and III) are acetyl-CoA, propionyl-CoA, and malonyl-CoA. They are involved in the biosynthesis of complex polyketides like pimaricin (Aparicio et al., 2000) or erythromycin (Cortés et al., 1990), among hundreds of macrolides and other polyketides. Protein acylation modulates the activity of enzymes involved in the biosynthesis of polyketide precursors, e.g., enzymes involved in the biosynthesis and the feeding of propionate, a precursor in erythromycin biosynthesis (Guo et al., 2016).

The propionylation of enzymes involved in erythromycin precursor biosynthesis in *Sac. erythraea* has been recently demonstrated. Using quantitative proteome analysis with anti-acetyl lysine and anti-propionyl lysine antibodies, Xu J. Y. et al. (2018) found 488 propionylation sites (Kpr) in 271 proteins in the proteome of *Sac. erythraea* and 631 lysine acetylation sites (Kac) in 366 proteins; this study reveals a large propionylome set in *S. erythraea.* Proteins containing more than one propionylation site include the elongation factors G and Ts, the fructose bisphosphate aldolase, and several chaperone proteins that play key roles in protein synthesis and regulation. Interestingly, some of the propionylation sites in these proteins were also acetylated or malonylated indicating that protein modification by lysine acylation is important for controlling cellular metabolism. The intracellular concentration of the different acyl-CoA molecules determines the selection of the acylating unit in *Sac. erythraea*; thus, the wealth of propionylation reflects the high pool of propionyl-CoA (Xu Y. et al., 2018). Several enzymes of the TCA cycle and the central carbon metabolism are propionylated in their lysine residues, including enzymes encoded by genes of the methyl malonate semialdehyde *mmsA* operon, involved in the intracellular synthesis of propionate. Lysine residues acylation occurs in the acyl-CoA dehydrogenase, the methyl-malonyl aldehyde dehydrogenase and the enoyl-CoA hydratase, all belonging to the *mmsA* cluster. Six of the lysine residues in the methyl-malonyl aldehyde dehydrogenase are acylated; and three of them are frequently propionylated. It was proposed that heavy propionylation of these enzymes reduce the formation of propionate and therefore decreased the biosynthesis of erythromycin in wild type *Sac. erythraea* strains (Xu J. Y. et al., 2018). These authors confirmed that the propionylation intensity is higher in low producing strains than in a high erythromycin producing mutant.

Malonylation of lysine residues rather than propionylation appears to significantly affect the biosynthesis of precursors for polyketides in some *Streptomyces* species, e.g., in *S. coelicolor*. For example, the polyketide-derived undecylprodiginine (the red pigment) synthesized by the *red* cluster of *S. coelicolor* is formed by a series of activation and condensing enzymes typical of the type II polyketides. Malonyl-CoA is used for the biosynthesis of undecylprodiginine. The question is, if there is a correlation between lysine malonylation of the malonyl-CoA biosynthetic enzymes and undecylprodigionine biosynthesis. Indeed, malonylation of the enzymes involved in the formation of prodigionine precursors controls the prodiginine biosynthetic enzymes activity and, thus, the red pigment formation (Xu J. Y. et al., 2016). In summary, all available evidence indicates that many enzymes involved in polyketide precursor formation are modified by post-translational acetylation, propionylation and/or malonylation of their lysine residues. The lysine acylation by high levels of some acyl-CoA molecules may be considered a feed-back control mechanism of the biosynthesis of specialized metabolites derived from these precursors. This hypothesis is supported by observations that deacylation of lysine residues by the SIRT deacylases improves in many cases the biosynthesis of secondary metabolites (You et al., 2016).

### Acylation of Transcriptional Factors and Other Key Enzymes in Actinobacteria Metabolism

So far post-translational lysine acylation of proteins has been focused on the modification of enzymes involved in the biosynthesis of secondary metabolites precursors. However, a significant finding is that several transcriptional factors, e.g., PhoP, GlnR, AfsR, the iron regulator FurR, or key enzymes in the regulation of *Streptomyces* and other actinobacteria metabolism, e.g., glucokinase, GlK, glutamine synthetase, GS, or SAM synthetase are all modified by lysine acylation (Table 2).

**TABLE 2:** Representative transcriptional factors and key regulatory proteins modified by acylation.

**Protein**	**Function**	**Strain**	**References**
GlnR	Global nitrogen metabolism regulation	*S. coelicolor*	Amin et al., 2016
PhoP	Phosphate control response regulator	*Sac. erythraea*	Xu Y. et al., 2018
AfsR	Large OmpR-like transcriptional regulator	*S. roseosporus*	Liao et al., 2014
AtrA	Transcriptional regulator	*S. roseosporus*	Sun et al., 2020b
Glk	Glucokinase, regulation of carbon catabolite	*S. roseosporus*	Sun et al., 2020b
GntR	Transcriptional Regulatory family	*S. roseosporus*	Liao et al., 2014
ArpA	γ-Butyrolactone receptor protein	*S. roseosporus*	Sun et al., 2020b
RelA	ppGpp synthetase	*S. roseosporus*	Sun et al., 2020b
MetK	S-adenosyl methionine synthetase	*Sac. erythraea*	Xu J. Y. et al., 2018
RpoA, B, C	RNA polymerase subunits	*S. roseosporus*	Liao et al., 2014
MarR	EPS-associated transcriptional regulator	*S. roseosporus*	Liao et al., 2014
CRP	cAMP receptor protein	*S. roseosporus*	Liao et al., 2014
Mihf	Histone-like protein	*S. roseosporus*	Liao et al., 2014

### Modification of the PhoP Global Regulator by Propionylation

The DNA binding domain of many response regulators of the OmpR-family frequently undergoes modification by post-translational acylation (Luo et al., 2004; Matsuzaki et al., 2005; Qin et al., 2016), affecting its DNA binding affinity. For example, mass spectrometry analysis revealed that the PhoP response regulator of *Sac. erythraea* is propionylated at lysines K^198^ and K^203^ (Xu Y. et al., 2018). The involved acyl transferase, encoded by *acuA*, normally uses acetyl-CoA as substrate, but in the PhoP acylation uses propionyl-CoA. *In vitro* mutation of K^198^ and K^203^ to the basic amino acid arginine still maintains most of PhoP^DBD^ binding ability, as demonstrated by EMSA studies. However, when the two lysines were mutated to glutamate or glutamine it resulted in weak binding of PhoP to the PhoP binding boxes in the DNA and no mobility shift was observed (Xu Y. et al., 2018). This behavior suggests that propionylation of PhoP results in a relaxed control of expression of the genes in Pho regulon. In parallel, propionylation of PhoP results in deregulation of the genes for secondary metabolism, which is generally controlled by the phosphate level in the medium. No information on the acylation of other PhoP response regulators of *Streptomyces* is known.

### Acetylation of the Global Regulator GlnR of *Streptomyces coelicolor*

In addition to modification by phosphorylation (see above), GlnR of *S. coelicolor* is acetylated at four lysine residues in complex medium [K^142^, K^153^, K^159^, K^200^ but only one residue [K^142^] is acetylated in a nitrogen-defined medium] (Amin et al., 2016). The post-translational modification of GlnR was confirmed by LC-MS/MS analysis of the isolated *S. coelicolor* GlnR protein; two of the acetylated lysine residues (K^153^ and K^200^) are located in the α-helix of this class of regulators and, therefore, might affect the expression of GlnR nitrogen-regulated genes. However, lysine acetylation of GlnR does not affect the binding of this regulator to the consensus GlnR binding sequences in the upstream regions of the well-known nitrogen metabolism genes. This response indicates that lysine acetylation of GlnR does not influence the transcriptional control of the nitrogen regulation. Still, it may play a role in the coordination of carbon and nitrogen metabolism. From the above discussed results, it is clear that both phosphorylation and acetylation of transcriptional factors might be performed in the same protein and in some cases, there might be overlapping.

There is very little information on the acylation of other transcriptional factors in *Streptomyces.* For instance, acetylated lysines were found in the histone-like protein MIHF and at the residue K^269^ of AfsR that corresponds to an ATP-binding domain. Thus, it is likely that acylation affects the regulatory role of this factor (Liao et al., 2014).

### Crotonylation of Lysine Residues of the Glucokinase and Its Effect on Carbon Catabolite Regulation

The presence of carbon sources with efficient assimilation like glucose promotes rapid microbial growth. However, it simultaneously prevents other carbon source’s catabolism by a mechanism called carbon catabolite repression (CCR) (Simpson-Lavy and Kupiec, 2019). This mechanism is one of the most conserved ones reported in various bacteria, including actinobacteria. It protects the cells against wasting protein-synthesizing machinery and controls secondary metabolism (Romero-Rodríguez et al., 2016, 2017; van Der Heul et al., 2018). In *Streptomyces*, CCR by glucose appears to be due to both carbohydrate metabolism intermediaries like fructose 1,6-bisphosphate or glucose 6-phosphate (Ramos et al., 2004; Borodina et al., 2008) and the enzyme glucose kinase (Glk) (Angell et al., 1994). Glk is the first step for glucose catabolism since it catalyzes glucose phosphorylation, resulting in glucose 6-phosphate (Kawai et al., 2005). Hodgson (1982) initially observed the relationship between Glk and CCR in mutant strains of *S. coelicolor* resistant to the glucose analog 2-deoxyglucose (2-DOGR) that also were insensitive to glucose repression. In the CCR mechanism, besides the possible effect of phosphoryl metabolic intermediaries (Ramos et al., 2004), Glk can also interact with transcriptional regulators (Romero-Rodríguez et al., 2017) or be subject to post-translational modifications by crotonylation by acyltransferases (Macek et al., 2019; Sun et al., 2020b). Among the amino acids targeted by these modifications, lysine plays an important role. Lysine crotonylation is structurally and functionally different from the widely studied amino acid acetylation. Its crotonylation depends on the dynamic balance between crotonyl-transferases and decrotonylases (Zhao et al., 2018).

*Streptomyces* Glk can be crotonylated, and this effect is reversed by the activities of decrotonylase CobB and the crotonyl-transferase Kct1 to negatively control its activity (Sun et al., 2020a). Furthermore, crotonylation positively regulates CCR for *Streptomyces* metabolism by modulating the ratio of glucose uptake/Glk activity and allowing other carbon sources utilization. Based on MS/MS data, Sun et al. (2020b) identified crotonylation of Glk at two conserved lysine residues (K^89^ and K^91^), adjacent to a catalytic aspartic acid (D^105^). Furthermore, they detected no acetylation of the Glk, excluding its influence on Glk as a possible target. Crotonylation inhibits the Glk activity, then the crotonylation and enzyme activity relation supports this effect as one of the post-translational modification mechanisms for eliciting the Glk regulatory effect. This mechanism is crucial to many microbes, including actinobacteria, enabling these microorganisms to rapidly respond to environmental changes, balancing the flux of several metabolic pathways.

## Acylation of Key Enzymes in the Metabolism of *Streptomyces*

Acetylation affects hundreds of enzymes in actinobacteria and other microorganisms. Some of the modified enzymes play pivotal roles in the biosynthesis of intermediates of secondary metabolites and/or their regulation. A few of the enzymes modified by acetyl transferases are described below.

### Glutamine Synthetase

Two of the glutamine synthetases in *Sac. erythraea* are named GlnA1 and GlnA4. The AcuA acetyltransferase acetylates GlnA4 inactivating its glutamine synthetase activity but has no significant effect on GlnA1 activity. However, partial acetylation of GlnA1 exerts an important effect by increasing its affinity to the global nitrogen regulator GlnR (You et al., 2016). As indicated above, the nitrogen regulator, GlnR, activates the *acuA* gene, encoding the acetyltransferase. Therefore, acetylation of GlnA1 increases expression of *glnR*, providing a feed-back loop mechanism that modulates the influence of nitrogen sources on cell metabolism. Comparative analysis of several actinobacteria suggests that the acetylation of these two glutamine synthetases is conserved throughout the actinobacteria.

### S-Adenosyl Methionine Synthetase

Another important enzyme that plays a key role in the one carbon transfer reactions is the S-adenosylmethionine (SAM) synthetase encoded in *Streptomyces* by the gene *metK* (Xu J. Y. et al., 2018). SAM is the key methyl group donor in microbial metabolism and plays an important role in the biosynthesis of erythromycin in *Sac. erythraea* and other methylated secondary metabolites (Wang et al., 2007). The *Sac. erythraea* SAM synthetase is propionylated at lysine K^274^. Xu J. Y. et al. (2018) concluded that high levels of propionylation of SAM synthetase reduce erythromycin production. Indeed, it seems that deficient levels of propionylation of this enzyme occur in erythromycin high-producing strains compared with the wild type low producer. Also, the SAM synthetase of other *Streptomyces* sp. conserves the K^274^ residue (Xu J. Y. et al., 2018); this lysine residue is important in this enzyme activity in *E. coli* (Taylor and Markham, 2000) and also in *Sac. erythraea.* On the other hand, as indicated above, the cellular SAM concentration appears to be sensed by the proteins AfsK/AfsR. The phosphorylation degree of these two proteins plays a key role in the methionine signaling cascade (Horinouchi, 2003; Jin et al., 2011; Martín and Liras, 2020).

## Post-Translational Modifications of Non-Ribosomal Peptide Synthetases, Long acyl-CoA Synthetases, and Other Enzymes for the Biosynthesis of Secondary Metabolites

So far, it is clear that post-translation protein lysine acylation is involved in the regulation of polyketide precursor formation. However, until recently, it was unclear whether the high molecular weight polyketide synthases themselves or the non-ribosomal peptide synthetases, involved in the biosynthesis of many natural products, are post-translationally modified by this mechanism.

### Non-ribosomal Peptide Synthetases

Acetylation of a lysine residue of a NRPS of *S. roseosporus* was reported by Liao et al. (2014), although the peptide product synthesized by this NRPS is unknown. Acetylation occurs at K^703^, located in an aminoacyl activation domain (domain A) of the NRPSs. The position of K^703^ in the A domain of the NRPS of *S. roseosporus* is equivalent to a conserved lysine residue in the phenylalanine-activating domain of the gramicidin synthetase in *Bacillus*. This lysine residue is essential for efficient adenylate formation, and its mutation blocks gramicidin biosynthesis. So far, a correlation between the acetylation of this lysine in the A domain with the activity of the NRPS *in vivo* has not been studied in *S. roseosporus.* The amino acids activation in the amino acid binding pocket of the NRPSs is made by the initial formation of aminoacyl adenylates using ATP (Figure 1B). The amino acyl group is then transferred to the -SH group of the phosphopantetheine moiety. There are many A domains in NRPSs that activate amino acids (Stachelhaus et al., 1999; Challis et al., 2000; Prieto et al., 2011) sharing this molecular mechanism. However, it is unclear if the acetylation occurs in all amino acid binding sites or whether it is specific for the activating domains of selected amino acids, e.g., the phenylalanine activating domains. The first step of the amino acid activation at the A domains of the NRPSs occurs also in the medium and long fatty precursors activation by acyl-CoA synthetases and in the luciferase (Conti et al., 1996; Marahiel, 2016). This activation mechanism is similar to that catalyzed by acetyl-CoA synthetase first-half reaction. Therefore, the acetylation at lysine residues at the A domain is likely to affect the activation of the different amino acids or fatty acids substrates in a variety of NRPSs but probably not in PKSs (see below).

### Long Chain Acyl-CoA Synthetases

As described above, early studies on protein lysine acetylation in *Streptomyces* allowed the discovery of acetyl-CoA and acetoacetyl-CoA synthetases acetylation. Later, Liao et al. (2014) observed that acetylated lysine residues occur in long chain acyl-Co synthetases at similar positions than the K^517^ in the amino acyl domain of adenylate-forming enzymes that plays similar role in the biosynthesis of fatty acids and some polyketides (Table 3). The exact physiological implication of the acetylation of long chain acyl-CoA synthetases in the biosynthesis of natural products derived from these fatty acids has not been described so far and deserves a detailed investigation. Surprisingly no reports on the acylation of PKSs are known. The lack of lysine acylation of PKSs correlates well with the fact that these enzymes use pre-formed malonyl-CoA or methylmalonyl-CoA as elongation units that do not involve activation of the short fatty acid by ATP to form acyl adenylates (i.e., they are synthases). This situation contrasts with the NRPSs and long chain acyl-CoA synthetases that activates the free amino acids or fatty acids with ATP (e.g., they are synthetases). We propose to use the formation of acyl adenylate intermediates to distinguish the authentic synthetases from synthases since the designation of these enzymes has been unclear so far. It would be interesting to investigate this correlation in-depth to understand the molecular mechanism of enzyme inhibition by acetylation. Some complex PKSs, such as the candicidin synthase or the geldanamycin synthase that use, respectively, p-aminobenzoyl-CoA (Martín and Aparicio, 2009) or 3-amino-5-hydroxybenzoyl-CoA (He et al., 2006) as starter units, have specific enzymes encoded in their clusters to activate the precursor unit. These activating enzymes, which initially form p-aminobenzoyl-AMP or 3-amino-5-hydroxybenzoyl-AMP, would be targets for lysine residues acetylation.

**TABLE 3:** Post-translational modification of non-ribosomal peptide synthetases (NRPSs), PKSs, long acyl-CoA synthases, and other antibiotic biosynthetic enzymes.

**Enzyme**	**Product**	**Type of modification**	**References**
NRPS	Non ribosomal peptide (unknown structure)	Acetylation	Xu J. Y. et al., 2018
Acyl-CoA synthase	Medium and long fatty-acyl-CoA	Acetylation	Liao et al., 2014
DesD	Desferroxiamine	Acetylation	Liao et al., 2014
FrbH decarboxylase/aminotransferase	Phosphinic acid-derived compound	Acetylation	Liao et al., 2014
StrM deoxysugar epimerase	Streptomycin	Acetylation	Ishigaki et al., 2017;
PPTases of ACP, PCP domains of NRPSs PKSs and FASs domains*	Holoenzymes NRPSs, PKSs y FASs	Pantetheinylation	Wang et al., 2016; Ordóñez-Robles et al., 2017

*There are numerous PPTases that modify enzymes of the biosynthesis of secondary metabolites.

### Non-ribosomally Synthesized Siderophores of the Desferrioxamine Type

*Streptomyces* species synthesize different types of siderophores of which the most common are hydroxamates of the desferrioxamine family (Barona-Gomez et al., 2004; Flores and Martín, 2004; Challis, 2005). The desferrioxamines are synthesized by four enzymes (DesA, B, C and D) encoded by the *des* cluster which is controlled by the DmdR regulator (Flores et al., 2005; Tunca et al., 2007). The DesD-encoded protein has two domains. One domain at the C-terminal end encodes an iron transporter, while at the N-terminal end, the protein has a lucA/lucC domain. Two lysine residues, K^287^ and K^357^, are located at the lucA/lucC domain, being the K^287^ residue conserved in all the DesD orthologous proteins. Both lysine residues might be acetylated in *S. roseosporus* (Liao et al., 2014), and K^287^ appears to interact with a phosphate group in an ATP/GTP nucleotide; therefore, its acetylation may affect the reaction mechanism if the ATP/GTP is required for activation (Challis, 2005).

### Acetylation of an Enzyme Required for the Biosynthesis of Phosphinic Acid-Derived Compounds

**FIGURE 2:**
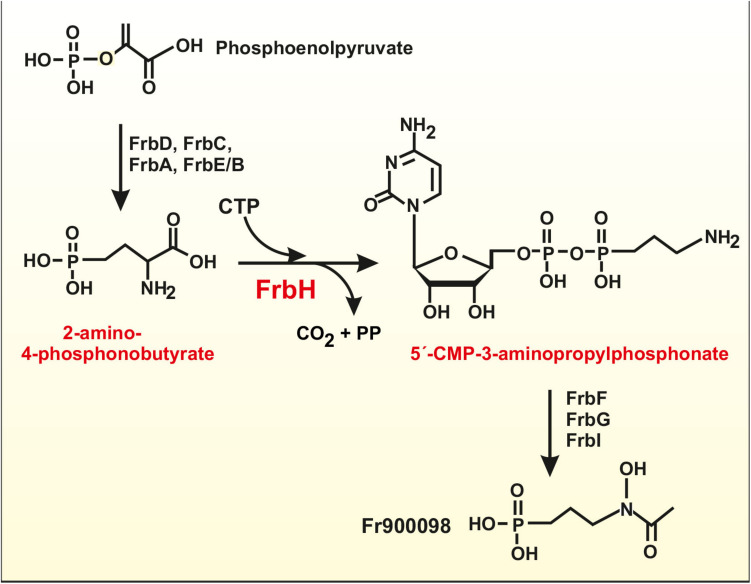
Schematic biosynthetic pathway of the antimalarial compound FR900098 in *S. ruberllomurinus* and *S. roseosporus*. The pyridoxal phosphate-dependent decarboxylase/aminotransferase reaction is performed by FrbH, an enzyme that is modified by acetylation at three different lysine residues. The reaction activates 2-amino-4-phosphonobutyrate with CTP and decarboxylate this compound forming 5′-CMP-3-aminopropylphosphonate. All the subsequent intermediates appear to be activated with CMP that is released at the final step of the pathway to give FR900098 (see text for details).

Some natural compounds that contain a phosphinic acid moiety are of interest because some of them have potent antimalarial and antibacterial activities, such as phosphomycin and phosphidomycin (Metcalf and van der Donk, 2009). A gene cluster, *frb*, encoding enzymes for one of these compounds, FR900098, produced by *Streptomyces ruberllomurinus* contains eight biosynthetic genes (*frbA-frbH*) and a resistance gene (Eliot et al., 2008; Metcalf and van der Donk, 2009). A similar cluster has been found in *S. roseosporus* (Liao et al., 2014). The protein encoded by *frbH* is a pyridoxal phosphate-dependent decarboxylase, which converts 2-amino-4-phosphonobutyrate in CMP-3-aminopropylphosphonate, including a decarboxylation reaction (Figure 2). FrbH has three lysine residues K^458^, K^568^, and K^573^, located in the pyridoxal phosphate-dependent aminotransferase domain. All these residues might be acetylated in the *S. roseosporus* orthologous protein, possibly affecting the enzyme activity (Liao et al., 2014). Interestingly, during the amino mutase reaction, 2-amino-4-phosphonobutyrate is activated with CTP forming CMP-3-aminopropylphosphonate (Figure 2), a reaction closely similar to those involved in the activation of amino acids in NRPSs. Therefore, the post-translational acetylation of the amino mutase may inhibit the formation of an amino acyl-adenylate-like molecule. Further studies are required to understand how this acetylation affects these antimalarial compounds biosynthesis.

### Acetylation of a Streptomycin Biosynthetic Enzyme in *Streptomyces griseus*

The acetylome of a streptomycin producer *S. griseus* strain was reported by Ishigaki et al. (2017). They observed that protein acetylation was induced in the secondary metabolites production and differentiation stage in liquid and solid cultures of *S. griseus.* The acetylome analysis revealed 134 acetylated proteins, most of them (58%) involved in primary metabolism and protein synthesis. The antibiotic streptomycin, is formed by the condensation of three moieties, namely streptidine, streptose, and N-methyl-α-L-glucosamine (Distler et al., 1992). One of the enzymes involved in its biosynthesis, encoded by the gene *strM*, was highly acetylated. The *strM* gene encodes a deoxysugar epimerase engaged in the formation of the streptose moiety. This isomerization reaction usually involves a deoxysugar-TDP intermediate (Wahl et al., 1975). The lysine K^70^ residue of the StrM protein is the major acetylation site in this protein, and remains conserved in deoxysugar epimerases. Mutation of the K^70^ residue to glutamine decreases streptomycin production drastically. However, indirect effects on the stability of the mutated deoxysugar epimerase may also contribute to this result.

*S. griseus* genome encodes a GNAT-type KAT that is 73% identical to PatA of *S. lividans*. Both the *S. griseus* lysine acetyltransferase and the orthologous *S. lividans* PatA protein, expressed in *E. coli*, and purified were able to acetylate StrM *in vitro* but not StrM proteins in which the K^70^ was changed to arginine, confirming that K^70^ is their specific target site. The authors propose that acetylation of the deoxysugar epimerase may reduce the biosynthesis of streptomycin in *S. griseus.*

## Post-Translational Modification of Proteins by Phosphopantetheneylation

A large number of enzymes involved in the biosynthesis of secondary metabolites undergo modification by the introduction of a phosphopantetheinyl group catalyzed by phosphopantetheinyl transferases (PPTases) (Lambalot et al., 1996; Mootz et al., 2001). In contrast to the modulating effect of phosphorylation and acetylation of proteins, the phosphopantetheinylation is an essential step in activating the substrate enzymes, and these include NRPSs, PKSs, fatty acid synthases, and some enzymes involved in primary metabolism, e.g., activation of amino acids and other intermediary metabolites in yeast and filamentous fungi. For example, the PPTase of *Penicillium chrysogenum* activates both the α-aminoadipyl-cysteinyl valine synthetase and the α-aminoadipate reductase involved in lysine biosynthesis (García-Estrada et al., 2008). The phosphopantetheinylation of these large enzymes converts the inactive (apo) enzyme into the active (holo) form. The introduction of the phosphopantetheinyl group by the PPTases takes place at the hydroxyl group of conserved serine residues by forming a phosphate-ester in the peptide carrier protein domains of NRPSs or the acyl carrier domain of fatty acid synthases and polyketides synthases (Walsh et al., 1997). Numerous PPTases have been identified in different bacteria, and they are structurally diverse (Wang et al., 2016). The PPTases are frequently encoded by discrete genes, clustered with other genes for PKS or NRPS, although occasionally the encoding genes are scattered in the genome. There are three types of PPTases: those of type I are formed by three subunits of about 120 amino acids (Huang et al., 2006; Dall’aglio et al., 2011). Type II is represented by the Sft-type PPTases consisting in basic polypeptides of about 240 amino acids (Sánchez et al., 2001; Meiser and Muller, 2008). Type III PPTases exist as integrated domains of polyketide synthases (Zhang et al., 2008) and usually phosphopantetheinylate the adjacent ACP domain of the polyketide synthases.

In contrast, discrete PPTases of class I or class II phosphopantetheinylate diverse ACPs or PCPs in different bacteria. The PPTases of actinobacteria modifies several proteins involved in primary and secondary metabolism (Weissman et al., 2004; Lu et al., 2008). Since there are different types of targets for PPTases, namely, ACP, PCP, and ACPs of fatty acid synthases a relevant question is, whether they are specific PPTases for each substrate domains. Another related question is whether the same PPTases are involved in the modification of enzymes of primary and secondary metabolism, e.g., the *P. chrysogenum* PPTase (García-Estrada et al., 2008). The information accumulated in the last decades shows that different classes of PPTases have a distinct degree of specificity. In some streptomycetes, e.g., *S. tsukubaensis* and *S. avermitilis*, there are up to five PPTases involved in the modification of different substrate proteins in the cell (Ordóñez-Robles et al., 2016, 2017; Wang et al., 2016). Three of the PPTases of *S. tsukubaensis* are engaged in the modification of the ACP domain of the polyketide synthase required for tacrolimus biosynthesis, while all fifth PPTases of this actinobacteria might modify the PCP domain, engaged in the integration of the pipecolyl-CoA moiety of tacrolimus (Ordóñez-Robles et al., 2016; Wang et al., 2016). Notably, one of these PPTases, Ppt1, is regulated by the global tacrolimus regulator FkbN (Ordóñez-Robles et al., 2017). Finally, genetic modification of PPTases encoding genes has been used to improve some antibiotic production (Jiang et al., 2013). In conclusion, when the substrate specificity of the PPTases is known, it is possible to improve the expression of a specific PPTase to increase the production of secondary metabolites containing the adequate ACP or PCP domain target.

## Protein Modification by Pupylation

Protein degradation of damaged or misfolded proteins is an essential part of the protein turnover to optimize amino acids utilization in the cells. In eukaryotic organisms, the proteins to be degraded are channeled to structures named proteasomes. In these structures, the proteins to be degraded are labeled by ubiquitination, a process in which the small protein ubiquitin is attached to the target substrate protein. In Gram-negative bacteria there are no proteasomes; thus, protein degradation takes place by proteolysis by ubiquitin-independent proteases. Ubiquitin-mediated degradation of proteins in proteasomes was believed to be exclusive of eukaryotic organisms. However, proteasomes were found in *S. coelicolor* (Nagy et al., 1998), and a prokaryotic ubiquitin-like protein (Pup) was discovered in *M. tuberculosis* (Pearce et al., 2008). In *Streptomyces* species, there are two different systems of protein degradation. One of them is proteasome independent, and the second one occurs in proteasomes. In prokaryotes, modification of the substrate proteins is named pupylation. Although this process seems to be frequent in actinobacteria, the knowledge of its molecular mechanism is still scarce (Burns and Darwin, 2010). Binding of the Pup protein in prokaryotes occurs in lysine residues of the targeted protein and requires the action of the PafA ligase (Pearce et al., 2008). In mutants lacking the PafA ligase there is no degradation of targeted proteins, establishing a correlation between pupylation and protein degradation. The Pup protein in *M. tuberculosis* is a small protein of 64 amino acids; at the carboxyl-terminal end Pup contains one glutamine (Pup-Q), which is deaminated to glutamic acid, forming the Pup-E biologically active variant. The ligation reaction mediated by PafA proceeds in two steps (Guth et al., 2011). In the first step, the carboxy-terminal glutamic acid is phosphorylated by ATP at carbon-5, by a mechanism similar to the formation of γ-glutamyl-phosphate in the glutamic acid derived amino acids biosynthesis, including glutamine. In the second step, the PafA ligase transfers the activated Pup-E to the target lysine residue. Pupylation is a reversible reaction as the modified proteins may be subject to depupylation by a depupylase activity located in the deaminase Dop (Burns et al., 2010; Imkamp et al., 2010).

Numerous proteins targeted by the pupylation systems have been identified in *M. tuberculosis*, *M. smegmatis*, *Corynebacterium glutamicum*, *Rhodococcus erythropolis*, and *S. coelicolor* (Akhter and Thakur, 2017). Metabolic studies of the pup-targeted proteins reveal that they play various physiological functions. Particularly affected are proteins involved in carbon catabolism, fatty acid metabolism, glycolysis, citric acid cycle, purine metabolism, and amino acid biosynthetic pathways (Akhter and Thakur, 2017). In *S. coelicolor* only the Pup-E variant is found and, therefore, does not need to be deaminated as occurs in *Mycobacterium;* however, the *dop* gene is still present, perhaps due to its involvement in the depupylation. In *S. coelicolor* the genes encoding the proteins involved in the pupylation system have been identified and they are well expressed in all the *Streptomyces* development stages (Boubakri et al., 2015). In *M. tuberculosis* and *S. coelicolor*, the proteins targeted by the pup-system are greatly influenced by the environmental and nutritional stressing conditions (Poulsen et al., 2010), particularly the oxidative stress conditions (De Mot et al., 2007; Boubakri et al., 2015). In *S. coelicolor*, mutants deleted in the PafA protein had an altered differentiation, affecting the formation of spores, antibiotic production, and resistance to oxidative stress (Compton et al., 2015); however, the deletion of the proteasome genes has little effect on cell development and secondary metabolism, indicating that pupylation has an additional role other than protein targeting to the proteasome (Boubakri et al., 2015; Compton et al., 2015). In contrast to *Streptomyces* species, *C. glutamicum* lacks the genes for the formation of proteasomes but still forms normal levels of pupylated proteins, although the alternative role of the pupylated proteins has not been elucidated. Proteomic studies of wild type *S. coelicolor* and a *pafA* mutant using histidine-tagged Pup protein revealed that 110 proteins were pupylated (Compton et al., 2015). The number of pupylated proteins in *S. coelicolor* is higher than that reported in *Mycobacterium.* This difference may be due to the larger genome of *S. coelicolor*, about twice that of *Mycobacterium.* Approximatedly 18% of the pupylated proteins were involved in regulation, and this may affect secondary metabolism. About 5% of those proteins were involved in stress and toxin resistance (Compton et al., 2015).

The available information indicates that pupylation affects actinorhodin, undecylprodigionine, and calcium-dependent antibiotic (CDA) production. However, a detailed study of antibiotic gene expression is required to clarify which regulatory factors or biosynthetic enzymes are affected (De Mot et al., 2007; Compton et al., 2015). With exception of *Streptomyces hygroscopicus*, depupylation systems have not been studied in other *Streptomyces* species. The pupylation system components from *S. hygroscopicus* have been expressed in *E. coli* (Xu X. et al., 2016) and *in vitro* studies allowed to characterize the *S. hygroscopicus* target proteins. These authors also confirmed the depupylation activity of the Dop deaminase in *S. hygroscopicus;* still, there are no reports on the effect of the pupylation system on secondary metabolites formation by S. *hygroscopicus.*

## Conclusion and Future Outlook

In conclusion, we now have a solid ground to build-up additional evidence in actinobacteria to identify the role of post-translational modifications in the fine-tuning of the metabolism regulation. However, there are still many obscure points in the control mechanisms of gene expression by modifying transcriptional factors that need investigation regarding future perspectives. The substrate protein specificity of the different post-translational modification systems also needs detailed elucidation. For instance, is the well-known *Streptomyces* acetyltransferase AcuA the only enzyme responsible for protein acylation in the presence of other acyl-CoA donors? Particularly intriguing is the use of bulky acyl-CoA molecules such as succinyl-CoA, crotonyl-CoA, or glutaryl-CoA that require a large binding pocket in the corresponding acyltransferases. The available evidence indicates that some of these enzymes are acyltransferases that may use different acyl donors (rather than acetyltransferases). However, additional acyltransferases encoded by duplicated genes with distinct substrate specificity may be involved. It is also important to clarify the acetylation involvement in the inhibition of the first half-reaction of the acyltransferase, which requires the formation of acyl-AMP intermediates in all documented cases. However, it is not entirely clear whether there are exceptions to this rule.

The formation of acyl-adenylate intermediates is characteristic of NRPSs and short and long chain fatty acyl-CoA synthetases. However, so far, there are no reports on the formation of acyl-adenylate intermediates by the modules of standard polyketide synthases and the fatty acid synthases. These two types of enzymes use preformed acyl-CoAs and do not need ATP for activation of elongation units. Therefore, they have been designated synthases in contrast to those enzymes that require ATP to form acyl-adenylate and are named synthetases. This observation needs to be supported by additional research to elucidate if any of the complex PKSs, or fatty acid synthases have domains to activate the starter units as acyl-adenylates. However, the evidence for enzymes such as the candicidin PKS (Martín and Aparicio, 2009), the rapamycin/tacrolimus PKS (König et al., 1997) or the geldanamycin PKS (He et al., 2006) indicates that enzymes encoded by discrete genes, usually clustered together with the polyketide synthase or the fatty acid synthase genes, achieve formation of the precursor units in those cases.

Some complex PKSs-NRPSs contain discrete genes encoding NRPS-like activating modules that use aminoacyl adenylate to integrate the acyl group in the growing polyketide chain, as is the case of the *rapP* and the *fkbP* genes in *Streptomyces hygroscopicus* and *S. tsukubaensis*, producers of rapamycin and tacrolimus. Another critical question that must be clarified is the scarce post-translational modifications observed in large multimodular enzymes involved in the synthesis of secondary metabolites and other bioactive compounds. There is no reason to believe that those enzymes would not be post-translationally modified as occurs with most other enzymes in bacterial metabolism. An exception is the post-translationally modification by phosphopantetheneylation performed by PPTases that has been investigated in several actinobacteria. However, the scope of phosphopantetheneylation needs to be expanded to other *Streptomyce*s species and particularly to actinobacteria other than *Streptomyces.* In the next future, the optimization of PPTases gene expression should be emphasized to improve the production of bioactive metabolites.

Regarding the outlook for pupylation studies, so far, they have been mainly focused on protein pupylation analysis in *M. tuberculosis*, owing to the importance of these modifications on its pathogenicity. Information on pupylation is scarce in *Streptomyces* and other actinobacteria. Particularly relevant is the effect of pupylation on the turnover of enzymes involved in secondary metabolites’ biosynthesis and the resistance to antibiotics and other stressing agents. The short life of many secondary metabolites biosynthetic enzymes has been reported in numerous studies. Still, the reason for the degradation of these enzymes and the mechanism involved in this turnover is not well known. More detailed studies on the pupylation mechanisms in *Streptomyces* and other actinobacteria will shed light on ways to prolong the enzymes half-life involved in secondary metabolite production. Likely, this may result in improvement of their production.

## Author Contributions

JM, PL, and SS designed the review content and wrote the various sections of it. All authors contributed to the article and approved the submitted version.

## Conflict of Interest

The authors declare that the research was conducted in the absence of any commercial or financial relationships that could be construed as a potential conflict of interest.
